# Thickness of the Buccal and Alveolar Bones Overlying Central Incisors: A Radiographic Iraqi Study

**DOI:** 10.1155/2022/7226998

**Published:** 2022-02-09

**Authors:** Nuhad A. Hassan, Aseel S. Khazaal Al-Jaboori

**Affiliations:** ^1^Department of Oral Medicine, College of Dentistry, AL-Mustansiriyah University, Baghdad, Iraq; ^2^Department of Prosthetic Dentistry, College of Dentistry, AL-Mustansiriyah University, Baghdad, Iraq

## Abstract

**Background:**

Initial bone thickness has a substantial impact on the success of dental implant treatments. The objective of the current study was to analyze the thickness of the buccal and alveolar bone at the central incisors using CBCT in relation to gender and side to determine the anatomical features and choose the best implant treatment option for minimizing the surgical complications.

**Methods:**

One hundred CBCT images were investigated (50 females and 50 males, aged 20 to 50 years old). The buccal bone thickness and alveolar bone thickness were evaluated for right and left sides of each subject at three sites; C: crest (3 mm); M: middle (6 mm); A: apical (9 mm) from the cementoenamel junction.

**Results:**

The mean thickness of buccal bone was less than 2 mm on the incisors according to side and gender. Buccal bone thickness revealed a statistically significant difference between right and left sides at the apical point in both females and males with *p* values of (*p* ≤ 0.001) and (0.001), respectively. The buccal bone thickness displayed statistically significant differences between genders at all sites. The alveolar thickness demonstrated similar significant differences between genders except for the crest site.

**Conclusions:**

Iraqi participants had about 1 mm buccal bone thickness at 3 mm apical from the CEJ in right and left central incisors with a progressive rise in bone thickness to be less than 2 mm at the apex. Alveolar bone also showed the same increase in bone thickness from crest to apex. Bone thickness was greater in males than females. The present study provided valuable CBCT data on bone thickness of the esthetic maxillary region as a preoperative analysis for establishing an immediate implant treatment plan with aesthetically pleasing long-term outcomes.

## 1. Introduction

Missing teeth are commonly treated with dental implants. The initial stability of dental implants and their survival rates are directly associated with patients' osseointegration ability [1]. The patients usually desire that their missing anterior teeth be replaced as soon as possible, so that they can resume their normal lives without the psychological problem of being edentulous and so that their appointments are reduced. Therefore, immediate implants placed between 0 and 7 days after tooth extraction came into existence to overcome this problem [2]. Devorah Schwartz-Arad concluded in their literature review article that immediate dental implants have a high survival rate when placed into fresh extraction sockets, and when inserted 3–5 mm beyond the apex and close to the crest of the alveolar bone [3]. The morphology, width, height, and density of the alveolar bone must be determined to choose the right size of the implant and the placement angle [4].

Bone thickness of the alveolar bone anteriorly is critical for dental implant therapy in terms of the esthetic outcomes. After tooth extraction, there will be a significant reduction in the buccolingual and apical-coronal extent of the alveolar region, which may affect the placement of implant-supported crown in the esthetic zone [5].

Moreover, sufficient buccal bone thickness in the anterior maxillary region is crucial for proper implant positioning. The thickness of the labial plate of the maxilla especially of the central incisors was reported to be very thin, necessitating extreme caution when inserting an implant [6, 7]. Next to upper central tooth extraction and immediate implant placement, the facial bone that covers the roots of the tooth is prone to resorption more than the palatal plate, leading to the center of the ridge to move to the palatal direction [8]. The amount of resorption is really affected by the original bone thickness. The thinner bone will contribute to a greater bone loss, dehiscence, fenestration, and recession of the soft tissue, risking the aesthetics and success of the implant [9, 10].

As studies have shown variances in bone patterns in various people, it is extremely crucial to pick a site for the installation of dental implants before the treatment and to thoroughly evaluate the oral anatomy and the width of both the ridge and the alveolar crest [11].

Cone beam computed tomography (CBCT) is extremely important for evaluating the advantages of the bony components surrounding the teeth as compared to older techniques. This technology has the advantages of high resolution, being noninvasive, low radiation dosage, and being cost efficient [12]. CBCT images are the most used means of measuring buccal bone thickness (BBT) in comparison of gender, age, and tooth differences across different patients and among other factors [13, 14]. Vera et al. [15] stated that CBCT scans can identify the BBT and its condition in the concerned area as an indicator for prolonged success of the implant.

A number of researches have employed CT to investigate maxillary alveolar bone thickness in the skull, but it may be inefficient as only two-dimensional data are available [16]. Few investigations have been conducted on CBCT, practically in the Iraqi population. Moreover, the anterior maxilla is esthetically sensitive for immediate dental implants position because of its particular anatomy. The objective of the current study was to analyze the thickness of the buccal and alveolar bone at the central incisors using CBCT in relation to gender and side of an Iraqi sample to determine the anatomical features and choose the best implant treatment option for minimizing the surgical complications.

## 2. Materials and Methods

One hundred CBCT images were investigated (50 females and 50 males, aged 20 to 50 years old), who were referred from different departments needing CBCT for various dental treatments. For all research participants, the ethical principles established in the Helsinki Declaration of 1964 were implemented. The local ethical approval by the Scientific Committee of Oral Medicine Department was obtained (protocol 16; November 2020). Following their approval, all participants provided consent forms. The minimum sample size was estimated to be 45 patients in each group using G*∗*Power 3.1.9.7 software and data generated through our pilot study.

The CBCT scanning was done by Myray, Italy, FOV 8 × 8 cm, voxel size 0.3 mm, exposure time 9.34 s, kVp 75, mA 5. The CBCT scans clearly showed the front maxilla and central incisors. Teeth having a root canal, periapical lesion, periodontal disease, malformation, or treatment such as fillings, crowns, or bruising were eliminated from the research.

**Figure 1:**
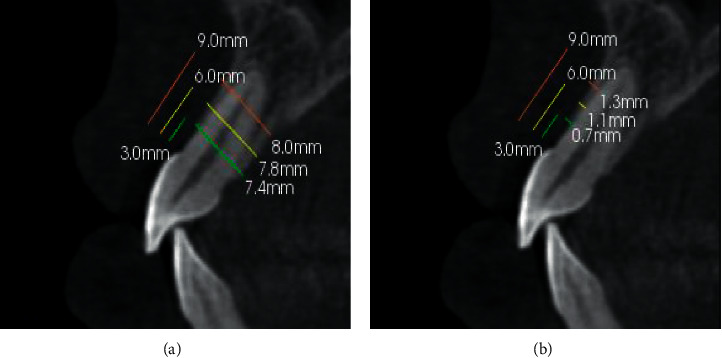
(a) Three locations for measuring alveolar bone thickness; (b) three sites for measuring buccal bone thickness.

The evaluation of the central incisors was done by CBCT. The data was collected and repeated by the same radiologist after one week. The two readings were almost the same, though to assess the intraobserver reliability, a paired *t*-test was conducted for 10 random CBCT scans showing no significant difference. The mean of each 2 measurements was utilized in the statistics. The buccal bone thickness (BBT) and alveolar bone thickness (ABT) were evaluated at three sites, which are C: crest (3 mm); M: middle (6 mm); A: apical (9 mm) from the cementoenamel junction (CEJ), and vertical to the long axis of the tooth at each point. The right and left sides were evaluated for all subjects [17], Figure 1.

### 2.1. Statistical Analysis

Data analysis was conducted with SPSS software version 21. Shapiro–Wilk and Levene's tests were used to investigate the data's normality and homogeneity of variance respectively. The independent *t*-test was used to compare thickness data between genders. A *p* value ≤0.05 was considered statistically significant.

## 3. Results

The study involved 100 CBCT images (200 teeth), divided equally into male and female groups.

The means of buccal bone thickness (BBT) at the crest, middle, and apical sites of females on the left side were (0.92 ± 0.22), (1.17 ± 0.16), and (1.19 ± 0.25), respectively. And they were (0.90 ± 0.25), (1.17 ± 0.20), and (1.47 ± 0.32) for the right side, respectively. For males, the means of BBT at the three sites were (1.00 ± 0.09), (1.20 ± 0.08), and (1.35 ± 0.21) for the left side, respectively. Likewise, they were (1.02 ± 0.08), (1.21 ± 0.06), and (1.48 ± 0.27) for the right side, respectively. Regarding the sides, the left BBT showed statistically significant difference across genders on two points (C and A), in spite of the right side showing statistically significant difference at one point only (C). The male groups had higher bone thickness than female groups at these points (Table 1).

In females, the mean thicknesses of the alveolar ridge (ABT) at the three sites (crest, middle, and apical) were (7.22 ± 0.44), (7.73 ± 0.30), and (8.44 ± 0.40) for the left side, respectively. Besides, they were (7.26 ± 0.43), (7.79 ± 0.42), and (8.50 ± 0.32) for the right side, respectively. Additionally, for males, the means were (7.24 ± 0.43), (8.24 ± 0.34), and (9.03 ± 0.47) for the left side, respectively, while they were (7.32 ± 0.40), (8.21 ± 0.36), and (9.10 ± 0.41) for the right side, respectively. The ABT on both sides was significantly different across gender (males showed higher bone thickness than females), except for the crest points (Table 2).

The *t*-test performed for comparison of the left and right sides of BBT by gender revealed statistically significant difference at only the apical points in both females (left side: 1.19 ± 0.25, right side: 1.47 ± 0.32, *p* ≤ 0.001) and males (left side: 1.35 ± 0.21, right side: 1.48 ± 0.27, *p*=0.001). The comparison between the left and right sides of ABT expressed significant differences at crest and apical sites only in males where the right sides showed a higher mean values with (*p*=0.032) and (*p*=0.019), respectively, Table 3.

In Table 4, the BBT displays statistically significant differences between genders at all sites. Bone thickness was greater in the males (crest: 1.01, middle: 1.205, apex: 1.415) than females (crest: 0.91, middle: 1.17, apex: 1.33). ABT demonstrated similar significant differences between genders except for the crest site.

Table 5 presents a statistically significant difference at the apical point for BBT concerning side, as bone thickness was increased at the right side (1.54 ± 0.34) compared to the left side (1.42 ± 0.34). Similar significant results were observed at the crest (right side: 6.11 ± 1.29, left side: 6.05 ± 1.29) and apical points (right side: 8.29 ± 1.15, left side; 8.21 ± 1.12) of ABT (*p* < 0.05).

The relationship between left and right BBT was highly statistically significant for the three sites (*r* = 0.52, *r* = 0.51 and *r* = 0.68), respectively (*p* ≤ 0.001). For ABT, the same statistically significant values were recorded (*r* = 0.98, *r* = 0.89, and *r* = 0.84), respectively.

## 4. Discussion

In dentistry, implant surgery is now the most prevalent therapy for lost teeth. Related research has shown that a positive bone state leads to greater osseointegration and an increased lifespan of dental implants; hence, a preoperative examination of the jawbone health is required [18, 19].

The current work applied CBCT to investigate bone thickness in the implantation sites as a presurgical assessment for the outer boney layer and the alveolar bone, which has proved to be useful for the patients in relevant studies using the same technology [20, 21]. CBCT has become the standard option for implant position planning, especially in esthetic zone. For example, augmentation surgical procedure may be required to enhance the bone around the dental implant if alveolar bone atrophy determined by CBCT was severe [9]. Compared to the palatal bone, the labial cortical plate at the anterior maxillary region is thinner, and resorption occurs more easily after teeth extraction [8]. The mid-buccal bone recession was about 0.5 mm after the first year of dental implantation according to a prospective study of single immediate implant placement at the maxillary anterior region [22]. In addition to that, excessive force applied to a thin buccal plate by implant insertion might cause microfractures and crestal bone loss. Therefore, it is critical to guarantee the existence of hard tissues to perform the implantation process without serious complications by using the right implant site and angling in the alveolar bone.

Moreover, the prevalence and rate of peri-implant soft tissue marginal recession that happens in the labial and interproximal sites after implant placement should be a major concern for clinicians from the dental implant aesthetics perspective. The mean free gingival recession around a single-tooth implant was found to be 0.5–1 mm [23]. Patients with thin scalloped gingiva were shown to be more prone to recession, whereas the thick-flat biotype was found to be a significant determining factor in achieving a satisfying esthetic outcome in implant restorations [24]. Further, certain periodontists have observed that thin alveolar contours are covered by thin gingival forms, suggesting that gingival contours are associated with underlying bone anatomy. As a result, soft tissue aesthetics surrounding implants are primarily influenced by bone anatomy [25].

With a number of modern studies [26, 27], the current work conducted on data to evaluate the thickness surrounding natural dentition. The soundness of determining BBT over natural teeth by CBCT has been approved and applied by many studies to find the length between CEJ and crest of the bone plus BBT of anterior teeth [28]. When assessing the thickness of the labial walls, choosing a reference point appears to be crucial. In the present study, CEJ was used as a reference point in accordance with previous studies using 3 mm distance [17, 29]. Measurements of bone thickness were also performed at 1 to 5 mm apical to the labial bone crest [13].

Clinicians have a public opinion that 2 mm is the smallest required thickness of the buccal wall to preserve the length of the alveolar crest and to calculate the quantity of crest resorption vertically after extraction [30]. Thus, due to the esthetic effect and long-term repercussions, the BBT must be carefully considered prior to tooth extraction and implant insertion. According to a one-year follow-up study, labial plate of 1-2 mm was suggested for better esthetic outcomes in immediate implant treatment, because enormous gingival recession and bone resorption were found in patients with a thickness of less than 0.5 mm [31]. Thus, in cases with thin buccal bone walls, dentists must consider the use of bone augmentation or graft material in immediate implant treatment plan [32]. In the current work, the mean of BBT on the left side was increased from 0.92 mm at the crest to 1.19 mm at the apex in females, while it was 0.90 mm coronally and 1.47 mm apically on the right side. According to males, it was 1.00 mm at the crest and increased to 1.35 mm at the apex of the left side. As for the right side, it was 1.02 mm coronally and 1.48 mm apically.

Januário et al. and other authors [13, 33] observed that BBT was slim and rarely >2 mm at different distances from CEJ during their studies by CBCT, which was compatible with the current outcome. Another study looked at BBT for 73 individuals without recording results exceeding 2 mm in bone thickness [30]. However, the finding was in disagreement with dos Santos et al. [34] who noticed that BBT was <1 mm in the majority of patients through examination of 202 CBCT (1463 teeth), while, in another prior study using 50 CBCT scan of maxilla, less than 10% of buccal bone sites had more than 2 mm thickness and in 14.4% of the central incisor buccal bone sites were ≥2 mm [35]. This variation in BBT results may be related to the difference in sample sizes, age, and gender.

The present work revealed a progressive rise in the thickness of the buccal and alveolar bone from the crest towards the apex. Koç et al. [36] and authors of other studies [37, 38] concluded a coinciding view with the present finding, indicating a steady increase in thickness in an apical direction. The finding was also consistent with that of Deguchi et al. [39], who discovered that the thickness of cortical bone increases with height and decreases with depth. For central incisors, the BBT was thicker at the apex and thinner at the crest. This provides insight into the thickness, reducing the risk of implant complications, as stated by Prakash et al. [40]. Another publication mentioned that the alveolar width of the central incisor diminished from the apical to crest direction [29], which is in line with the current work. In contrast, AlTarawneh et al. [41] reported that the mean measure of labial plates decreased towards the apical portion. This discrepancy might be attributed to the characteristics of the included cases as well as the different measuring depth used.

The absence of significance in the current study among BBT on the left and right sides was observed for the first two points (Table 3) since only the apical point was significant. Lee et al. [29] found no significant difference between the left and right BBT through a study of twenty participants.

The present study described the statistically significant difference of the left BBT between genders on two points (C and A), in spite of the right BBT being statistically significant at one point only (C). Fuentes et al. [35] noted that the BBT of the right central did not vary by gender, in contrast to the left central that showed a significant difference by this factor, and proposed that gender is not an indicator for thickness since this exhibited a different impact by gender.

The current finding indicated that males possess larger BBT than females at various root levels regardless of sides. The result is supported by specific past study groups [26, 29, 42]. Additionally, Adiguzel et al. [43] concluded that gender was significantly related to the BBT in the maxilla. According to the Cassetta et al. [44] study on 48 computed tomography scans, men had thicker alveolar cortical bone than women, and the alveolar crest thickens as it approaches the apex. Age could also be a deciding factor according to the literature. It has been noted that the thickness of the labial bone at the cervical part decreases with age [45], and that postmenopausal women have much thinner labial bone [37].

This study concluded gender differences in thickness of the alveolar ridge that women had smaller thickness than men except for the crest site. Men had stronger muscles for mastication than women to apply higher biting force [46]. Uner et al. [17] conducted a study on 160 CBCT (320 teeth) at 3, 6, and 9 mm depths below the crest with ages ranging from 21 to 53 and suggested a gender difference. Males had wider alveolar ridges than females in every location studied, which approximated current work except for the crest, which was statistically nonsignificant. Likewise, Lim et al. [47] asserted that women had a smaller ridge width than men during CBCT study of 32 subjects (13 men; 19 women).

Resorption of the buccal plate is more likely to impact the anterior regions than the posterior ones because the resorption is extra serious when the walls are first thinner. CBCT is the optimal method for preventing compromises in esthetic reconstruction [48]. It enables multiple measures at several sites and the existent bone property [49]. The research had certain limitations, which included the age impact. Second, the study only covered Iraqi patients. Several outcomes may not apply to other ethnicities. Finally, when the bone plate is exceedingly thin, CBCT readings may produce substantial inaccuracies and overestimations.

## 5. Conclusions

The present study presents valuable information regarding the thickness of buccal walls in the esthetic maxillary central incisor region in an Iraqi population as a preoperative analysis for establishing immediate implant treatment plan for aesthetically pleasing long-term outcomes.

Iraqi participants had about 1 mm buccal bone thickness at 3 mm apical from the CEJ in right and left central incisors sites with a progressive rise in the thickness to be less than 2 mm at the apex. In the esthetic maxillary region, there is a significant preponderance of thin BBT, which should be considered when performing dental procedures, such as tooth extractions or immediate implants. Alveolar bone also showed the same increase in bone thickness from crest to apex. Bone thickness was greater in males than females.

## Figures and Tables

**Table 1:** Descriptive statistics of left and right buccal bone thickness (mm) by gender with comparison of thickness between males and females according to side using *t*-test.

	*N*	Mean		Std deviation		*p* value
		Female	Male	Female	Male	
Left crest	50	0.92	1.00	0.22	0.090	0.013^*∗*^
Left middle	50	1.17	1.20	0.160	0.080	0.252
Left apical	50	1.19	1.35	0.250	0.210	0.001^*∗*^
Right crest	50	0.90	1.02	0.25	0.080	0.003^*∗*^
Right middle	50	1.17	1.21	0.200	0.060	0.145
Right apical	50	1.47	1.48	0.320	0.270	0.986

*N* = number of sample, Std deviation: standard deviation, *p* = probability value, ^*∗*^Statistically significant (*p* < 0.05).

**Table 2:** Descriptive statistics of left and right alveolar bone thickness (mm) by gender with comparison of thickness between males and females according to side using *t*-test.

	*N*	Mean		Std deviation		*p* value
		Female	Male	Female	Male	
Left crest	50	7.22	7.24	0.440	0.430	0.827
Right crest	50	7.26	7.32	0.430	0.40	0.505
Left middle	50	7.73	8.24	0.300	0.340	≤0.001^*∗*^
Right middle	50	7.79	8.21	0.42	0.360	≤0.001^*∗*^
Left apical	50	8.44	9.03	0.400	0.470	≤0.001^*∗*^
Right apical	50	8.50	9.10	0.320	0.410	≤0.001^*∗*^

*N* = number of sample, Std deviation = standard deviation, *p* = probability value, ^*∗*^High statistically significant *p* ≤ 0.001.

**Table 3:** *T*-test between left and right bone thickness (mm) for both males and females by sites.

	*N*	*p* value	
		Female	Male
Crest buccal bone thickness	50	0.3770	0.3760
Middle buccal bone thickness	50	0.9920	0.3200
Apical buccal bone thickness	50	≤0.001^*∗∗*^	0.001^*∗∗*^
Crest alveolar bone thickness	50	0.2440	0.032^*∗*^
Middle alveolar bone thickness	50	0.2910	0.4030
Apical alveolar bone thickness	50	0.3290	0.019^*∗*^

*p* = probability value, ^*∗*^Statistically significant (*p* < 0.05), ^*∗∗*^High statistically significant.

**Table 4:** *T*-test of bone thickness (mm) between males and females according to site.

	*N*	Mean		*p* value
		Male	Female	
Crest buccal bone thickness	100	1.01	0.91	0.015^*∗*^
Middle buccal bone thickness	100	1.205	1.17	0.013^*∗*^
Apical buccal bone thickness	100	1.415	1.33	0.003^*∗*^
Crest alveolar bone thickness	100	7.28	7.24	0.505
Middle alveolar bone thickness	100	8.22	7.76	0.003^*∗*^
Apical alveolar bone thickness	100	9.06	8.47	0.004^*∗*^

*p* = probability value, ^*∗*^Statistically significant (*p* < 0.05).

**Table 5:** Descriptive statistics of bone thickness (mm) by side and comparison of left and right thickness according to site.

	*N*	Mean		Std deviation		*p* value
		Left	Right	Left	Right	
Crest buccal bone thickness	100	0.990	1.00	0.1970	0.2480	0.630
Middle buccal bone thickness	100	1.24	1.26	0.195	0.248	0.300
Apical buccal bone thickness	100	1.42	1.54	0.3420	0.3430	≤0.001^*∗∗*^
Crest alveolar bone thickness	100	6.05	6.11	1.297	1.291	≤0.001^*∗∗*^
Middle alveolar bone thickness	100	7.17	7.19	1.047	1.046	0.505
Apical alveolar bone thickness	100	8.21	8.29	1.127	1.157	0.047^*∗*^

*N* = number of samples, Std deviation = standard deviation, *p* = probability value, ^*∗*^Statistically significant (*p* < 0.05), ^*∗∗*^High statistically significant *p* ≤ 0.001.

## Data Availability

The data used to support the findings of this study are available from the corresponding author upon request.
